# Riedel’s Lobe and Beaver Tail Variant: A Cadaveric Case Report

**DOI:** 10.7759/cureus.54301

**Published:** 2024-02-16

**Authors:** Femina Sam, Jenny Jacob, Suganthy Rabi

**Affiliations:** 1 Anatomy, Christian Medical College, Vellore, IND

**Keywords:** abdominal mass, hepatic, accessory, liver, variations

## Abstract

Variations of the liver in the form of accessory lobes account for about less than 1%. One of the commonest accessory lobes on the right is Riedel’s lobe. An occasional anatomical variant of the left lobe is the beaver tail variant. Both variants are reported to be common in females. We hereby report two cases. A 45-year-old female cadaver was noticed to have Riedel’s lobe and beaver tail variant. Riedel’s lobe appeared to be a tongue-like projection from the right inferior margin of the liver, almost reaching the level of the iliac crest. It was partially separated from the rest of the liver by a deep fissure. The left lobe was elongated, crossed the midline, and reached the left wall of the abdominal cavity after encircling the spleen. Splenomegaly was noticed, and the left lobe of the liver had a splenic impression. Riedel’s lobe was drained by the middle hepatic vein (MHV) and supplied by the right branch of the portal vein (RPV). A similar variation of the beaver tail variant was noticed in an 85-year-old female cadaver. The left lobe of the liver crossed the midline and was related superior to the spleen. The right lobe of the liver and the spleen were normal. The beaver tail variant was drained by the left hepatic vein (LHV) and supplied by the left branch of the portal vein (LPV). These variations are prone to injuries and can be confused with the abdominal mass and could interfere with laparoscopic procedures.

## Introduction

Accessory liver lobes are a rare morphological condition, and it is due to excessive development of the liver [1]. Their form, location, and size show variability [2]. Their prevalence is less than 1% as they are mostly asymptomatic [3,4]. These accessory liver lobes are mostly found incidentally during laparotomy and radiological investigations [3]. Yet, there are reports of accessory lobes turning to carcinoma and a few leading to torsions published in the literature [1,3]. It is imperative to document rare findings like accessory lobes to facilitate future diagnosis. One such known variant on the right side is Riedel’s lobe which corresponds to the hypertrophy of segments V and VI [1]. The original definition of Riedel’s lobe is “a downward tongue-like projection of the right lobe of the liver to or below the level of the navel or if the liver is extended caudal to the most inferior part of the costal margin in CT images” [3]. Another variant, which was less commonly reported but on the left side, was the beaver tail variant, which was an elongated left lobe of the liver that extends across the midline and often surrounds the spleen [5]. The beaver tail variant is an incidental finding and is usually encountered during abdominal imaging. It is still very difficult to distinguish it from the spleen because of the similar echogenicity in radiological images [5]. When it comes to liver transplantation, if the left lobe of the liver has a larger volume, it is sufficient as the remnant liver for the donor [6]. Here, we report two cadaveric cases of Riedel’s lobe and beaver tail variant of the liver, incidentally observed during the routine abdomen dissection of cadavers for undergraduate medical students.

## Case presentation

Case report 1

**Figure 1:**
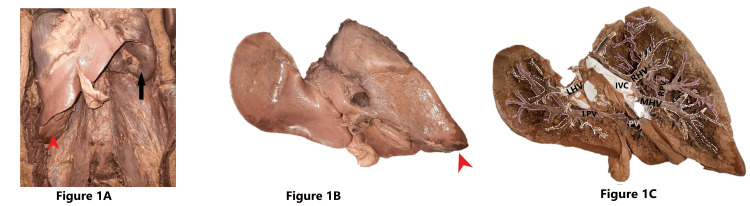
Cadaveric image of the liver with Riedel’s lobe and beaver tail variant Figure 1A shows Riedel’s lobe (red arrowhead) and beaver tail variant in the abdomen of a 45-year-old female cadaver. The black arrow indicates the spleen, forming an impression on the left lobe of the liver. Figure 1B shows the liver being removed from the abdomen. The red arrowhead indicates Riedel’s lobe. Figure 1C shows the portocaval intrahepatic structures of the liver. The hepatic venous radicals draining the liver into the IVC are depicted in white dotted lines and the portal venous radicals in purple dotted lines. Note that Riedel’s lobe is drained by the MHV and supplied by the RPV. The beaver tail variant is drained by the LHV and supplied by the LPV IVC: inferior vena cava; MHV: middle hepatic vein; RPV: right branch of the portal vein; LHV: left hepatic vein; LPV: left branch of the portal vein

**Figure 2:**
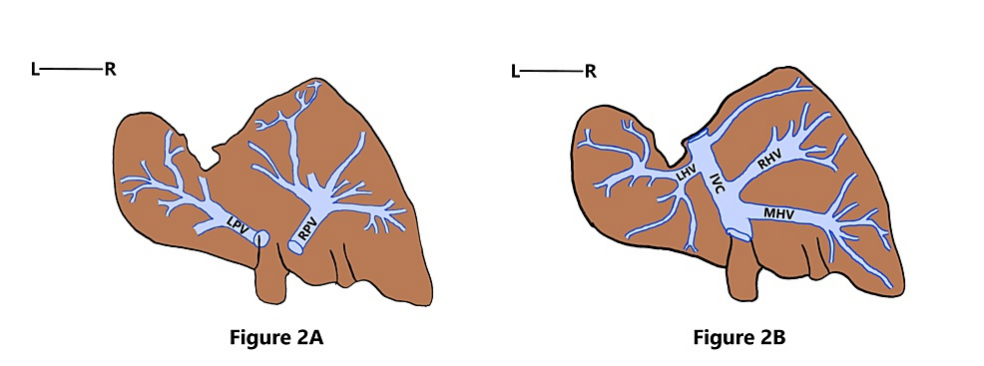
Schematic diagram showing the venous pattern of Riedel’s lobe and the beaver tail variant Figure 2A: Schematic diagram depicting the portal venous radicals supplying Riedel’s lobe and the beaver tail variant. Figure 2B: Schematic diagram depicting the hepatic venous radicals draining Riedel’s lobe and the beaver tail variant RPV: right branch of the portal vein; LPV: left branch of the portal vein, RHV: right hepatic vein; MHV: middle hepatic vein; LHV: left hepatic vein; IVC: inferior vena cava

A 45-year-old female cadaver was noticed to have Riedel’s lobe as well as a beaver tail variant. Riedel’s lobe extended from the right inferior margin of the liver as a downward elongated right lobe of the liver, having the same consistency and texture as the right lobe of the liver. It was found to almost reach the level of the iliac crest and was separated from the rest of the right lobe by a deep fissure. The beaver tail variant reached the left wall of the abdominal cavity after encircling the spleen. Splenomegaly was noticed, and the left lobe of the liver had a splenic impression (Figure 1A, 1B). The portocaval draining tributaries of Riedel’s lobe and beaver tail variant were looked for after injecting silicone into the lumen of both portal vein and hepatic veins and then by finger fracturing the parenchyma of the liver, after the immersion of specimen in acid. Riedel’s lobe received blood through the right branch of the portal vein (RPV) and was drained by the middle hepatic venous radical. The beaver tail variant was supplied by the left branch of the portal vein (LPV) and drained by the left hepatic vein (LHV) into the inferior vena cava (Figure 1C, Figure 2A, 2B).

Case report 2

**Figure 3:**
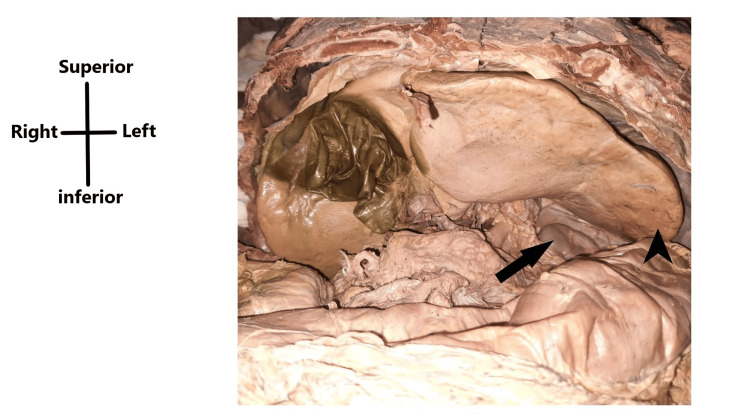
Cadaveric image showing the beaver tail variant of the liver within the abdomen The cadaveric image shows the presence of the beaver tail variant (black arrowhead) and the spleen (black arrow) being located inferior to the variant in case 2

While dissecting an 85-year-old female cadaver, the liver was noticed to have a beaver tail variant. The left lobe of the liver was elongated and crossed the midline as well as the left midclavicular line and extended to the spleen and was related superior to it, not completely encircling it. The right lobe of the liver and the spleen were normal (Figure 3).

## Discussion

Accessory liver lobes are defined as supernumerary liver lobes, composed of normal liver parenchyma in continuity with the liver. They can be congenital or acquired. Congenital variation was supported by different hypotheses like displacement of the primitive rudiment of the organ or by the persistence of mesodermal septa during proliferation of the liver anlage or by further branching of the foregut diverticulum [2,7]. Riedel considered it to be due to tractions exerted by the adherent cholecystitis. The first documentation dates back to 1830 by Corbin, and, in 1888, Riedel described a tongue-like elongation, to the right of the gallbladder [1]. It has been called a floating, tongue-like, and constriction lobe [2]. As liver segmentation described by Couinaud is concerned, Riedel’s lobe is described as an elongation of segments V and VI separated from the rest of the liver parenchyma by a groove [8]. While looking at the portocaval tributaries of Riedel’s lobe, the middle hepatic vein drains it, and it is supplied by the RPV.

The left lobe may also show a lot of variations in size and shape. Occasionally, an anatomical variant called the beaver tail variant was found, commonly in females. It resembles the tail of a beaver, hence the name. It consists of normal hepatic parenchyma in which the lateral part of the left lobe of the liver extends laterally to the spleen and sometimes even encircles it [9]. In case 1, the beaver tail variant encircled the spleen completely, and the splenic impression was noticed on the visceral surface of the left lobe of the liver. In case 2, the beaver tail variant proceeded superior to the spleen, without encircling it. This lobe can be considered as the elongated segment II, according to Couinaud’s segmentation of the liver. The beaver tail variant is drained by a left branch arising from the confluence of branches from the LHV and drained by the LPV. This beaver tail variant accounts for one of the pitfalls in computed tomography scan diagnosis, as it mimics perisplenic hematoma [10]. This variant should not be confused with appendix fibrosa hepatis, i.e., an atrophied fibrous band that attaches to the diaphragm at the left extremity of the liver and sometimes contains atrophied biliary ducts [11]. The beaver tail variant is more prone to injury in case of trauma occurring to the lower left chest or in the left upper quadrant of the abdomen [12]. Nevertheless, choosing living donors with the beaver tail variant may be safer in terms of remnant liver volume [6]. This variant is very useful, as post-transplantation liver failure in right lobe donors has been reported to be approximately 10% [6].

## Conclusions

It is imperative to know the variable morphological segmentation of the liver for surgeons. Knowledge of these morphological variants is of paramount importance before laparoscopic cholecystectomy as well as during liver transplantation surgeries.
